# Infectious disease diagnosis by artificial intelligence (AI): Differences in patient backgrounds and symptoms between antigen test positives and novel AI-powered pharyngeal endoscopy test positives

**DOI:** 10.1371/journal.pdig.0001233

**Published:** 2026-02-11

**Authors:** Masahiko Mori, Shinji Yoshinaga, Tadayoshi Moriyama, Takafumi Maekawa

**Affiliations:** 1 Department of Internal Medicine, Sasebo Memorial Hospital, Sasebo, Nagasaki, Japan; 2 Department of Medical Information, Sasebo Memorial Hospital, Sasebo, Nagasaki, Japan; 3 Department of Neurosurgery, Sasebo Memorial Hospital, Sasebo, Nagasaki, Japan; 4 Department of Surgery, Sasebo Memorial Hospital, Sasebo, Nagasaki, Japan; 5 Department of Surgery, Fukuoka Central Hospital, Fukuoka, Fukuoka, Japan; Brown University School of Public Health, UNITED STATES OF AMERICA

## Abstract

This study aimed to identify differences in patient background and symptoms between individuals who tested positive using conventional rapid antigen (Ag) tests and those who tested positive using a novel artificial intelligence (AI)–powered pharyngeal endoscopy system. A total of 813 patients underwent both influenza/COVID-19 Ag testing and AI-powered endoscopic testing. We analyzed differences in patient characteristics and symptoms between the two test-positive groups. AI testing showed an overall percent agreement of 62% (95% confidence interval [CI] 58–66%) (442/713), a positive percent agreement of 47% (95% CI 40–53%) (125/269), and a negative percent agreement of 71% (95% CI 67–76%) (317/444) compared with Ag testing. Compared with Ag-positive cases, AI-positive cases exhibited a shorter interval from symptom onset to testing; median 18 hours (Interquartile range [IQR] 10–27) for AI+ and Ag-, 24 hours (IQR 18–41) for AI+ and Ag + , and 27 hours (IQR 17–47) for AI- and Ag+ (p < 0.001). In analyses comparing the AI+ and Ag- vs. AI- and Ag + , AI+ and Ag- were more frequently paediatric (<15 years old) (odds ratio [OR] 3.4 (95% CI 1.6-7.2), p = 0.001), tested earlier after symptom onset (<24 hours) (OR 2.6 (95% CI 1.3-4.9), p = 0.005), had contact with infected individuals (OR 4.6 (95% CI 2.2-9.3), p < 0.001), cough (OR 11 (95% CI 4.7-27), p < 0.001), and fever (≥38.0°C) (OR 5.6 (95% CI 2.8-11), p < 0.001), but showed lower frequencies of gastrointestinal symptoms (OR 0.2 (95% CI 0.05-0.9), p = 0.04). Notably, the AI system misdiagnosed 23% (23/99) of COVID-19-positive patients as influenza-positive, likely due to follicular lesions on the pharyngeal wall—a key feature used by the AI system for diagnosing influenza. These findings demonstrate the impact of differences in diagnostic methodologies between conventional Ag testing (which detects pathogen viral load) and novel AI testing (which assesses host immune response to viral infection) on the clinical characteristics of test-positive patients.

## Author summary

This cross-sectional study investigated differences in patient backgrounds and symptoms between influenza-positive cases diagnosed by rapid antigen tests and those identified by a novel artificial intelligence (AI)-powered endoscopy system recently introduced in Japan. Compared with antigen test positives, AI test positives were tested earlier after symptom onset and were more frequently paediatric and symptomatic. These findings suggest potential utility of the AI testing system at the point-of-care, particularly for patients tested during the early phase of illness, when antigen test sensitivity is reduced. These findings highlight the impact of differences in diagnostic methodologies—namely, conventional rapid antigen testing, which detects pathogen viral load, versus the novel AI approach, which evaluates host immune response—on the clinical characteristics of test-positive patients. In addition, we observed cases in which the AI system misdiagnosed COVID-19 as influenza, likely due to symptomatic similarities between the two infections. Together, these findings underscore the promise of AI-based diagnostic systems for infectious diseases, while emphasizing the need for further deep learning-based improvements to enhance differentiation among respiratory pathogens.

## Introduction

During the COVID-19 pandemic from 2020 to early 2023, the number of seasonal influenza virus infections worldwide dramatically decreased but surged again after the pandemic subsided (WHO FluNet. https://www.who.int/tools/flunet). Similarly, in Japan, an influenza epidemic re-emerged after national COVID-19 prevention policies were lifted in May 2023 [1]. Influenza and COVID-19 share similar respiratory and systemic symptoms, making it difficult for clinicians to distinguish between the two pathogens based on clinical presentation alone, particularly since the emergence of the Omicron variant, which has been associated with smaller differences in disease severity between the two infections [2].

Throughout the COVID-19 pandemic, rapid antigen (Ag) testing using nasopharyngeal swab samples for viral infections, including COVID-19 and influenza, became widely recognized as a point-of-care diagnostic method in hospitals and drive-through testing centres [3]. However, concerns persisted regarding the discomfort caused by swab collection and the risk of viral exposure to healthcare workers, particularly when obtaining samples from paediatric patients [4].

As an alternative diagnostic method for influenza virus infection, an artificial intelligence (AI)-powered pharyngeal endoscopy system was introduced in Japan in December 2022 [5]. This system analyzes pharyngeal images captured by an endoscopic scope, enabling AI to detect follicular nodules on the posterior pharyngeal wall—findings commonly observed in patients with influenza virus infection [6]. By integrating clinical information such as patient backgrounds and symptoms, the AI system provides an automated diagnosis of influenza virus infection [5]. However, differences in diagnostic outcomes between this AI-powered pharyngeal endoscopy system and conventional rapid Ag testing—and the potential role of AI-based diagnosis in point-of-care testing in outpatient settings—remain unclear.

The primary objective of this study was to identify differences in patient backgrounds and symptoms between individuals who tested positive for influenza by conventional rapid Ag testing and those who tested positive using the AI system. A secondary objective was to explore challenges in developing AI-based diagnostic models for infectious diseases by examining cases misclassified by the AI system, with a particular focus on COVID-19 cases incorrectly diagnosed as influenza.

## Results

### Characteristics of subjects

The characteristics of sex, age, and influenza testing positivity among 813 subjects are presented in Table 1 and S1 Table (original dataset). In total, 52% (420/813) of patients were diagnosed with influenza virus infection as positive by either AI or Ag. Among these, 276 were positive by AI, and 270 by Ag. Of the 270 Ag-positive cases, 50% (134/270) were infected with influenza A strain and 50% (136/270) with influenza B strain. In addition, 12% (100/813) of patients were diagnosed with COVID-19 infection.

**Table 1:** Characteristics of study subjects.

n = 813			
Sex (female)	419 (52%)	Symptom	
Age (yo)^a^	14 (11-36)	Cough	548 (67%)
Paediatric (<15 yo) n = 409 (50%)	11 (9-12)	Sore throat	486 (60%)
Adult (≥15 yo) n = 404 (50%)	36.5 (21-53)	Headache	463 (57%)
History		General fatigue	446 (55%)
Contact with infected individuals	313 (38%)	Runny nose	442 (54%)
Use of antipyretics	347 (43%)	Fever (≥38.0°C)	381 (47%)
Influenza vaccination	226 (28%)	Chill	269 (33%)
Influenza virus testing		Appetite loss	213 (26%)
AI+ or Ag+	420 (52%)	Joint pain	184 (23%)
AI+	276 (34%)	Muscle pain	120 (15%)
Ag+	270 (33%)	Sweating	84 (10%)
Ag A+	134 (50%)	GI^b^ symptom	69 (8%)
Ag B+	136 (50%)		
COVID-19 Ag+	100 (12%)		

^a^ Median (interquartile range).

^b^ GI; Gastrointestinal.

**Fig 1:**
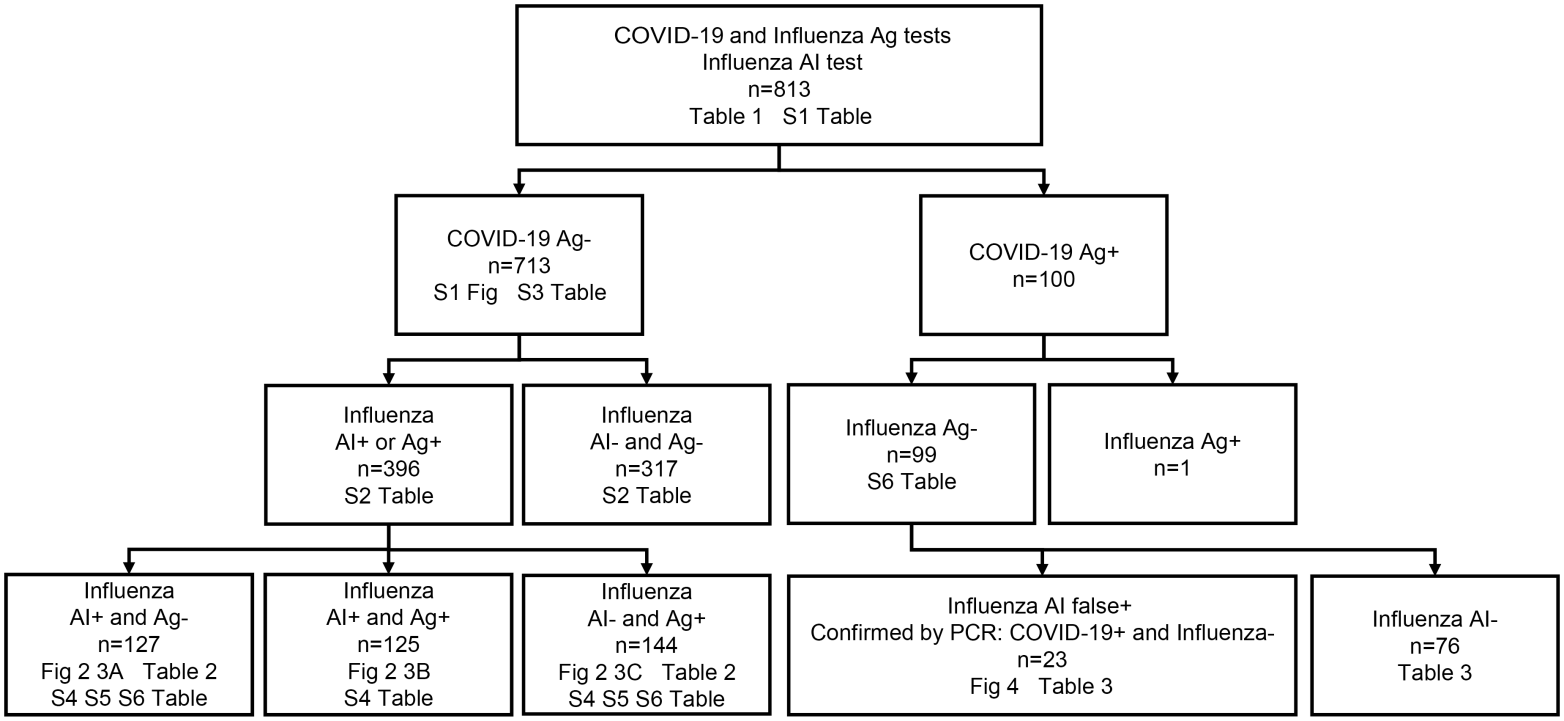
Participant flow diagram. The numbers of figures and tables whose analyses were performed are indicated.

Among the 713 COVID-19-negative cases (shown on the left side of the participant flow diagram in Fig 1), the 396 influenza testing-positive patients were younger (median 13 (interquartile range [IQR] 10–27) years vs.15 (IQR 11–34) years, p = 0.003), had a higher frequency of contact history with infected individuals (48% (189/396) vs. 24% (75/317), odds ratio [OR] 2.9 (95% confidence interval [CI] 2.1-4.1), p < 0.001) and more frequent use of antipyretics (45% (178/396) vs. 37% (118/317), OR 1.4 (95% CI 1.02-1.9), p = 0.04). They had a lower frequency of influenza vaccination (22% (89/396) vs. 31% (98/317), OR 0.6 (95% CI 0.5-0.9), p = 0.01). Symptomatically, they showed higher frequencies of cough (81% (322/396) vs. 40% (157/317), OR 4.4 (95% CI 3.2-6.2), p < 0.001), runny nose (64% (254/396) vs. 42% (134/317), OR 2.4 (95% CI 1.8-3.3), p < 0.001), fever (≥38.0°C) (65% (259/396) vs. 31% (98/317), OR 4.2 (95% CI 3.1-5.8), p < 0.001), joint pain (24% (95/396) vs. 17% (55/317), OR 1.5 (95% CI 1.04-2.2), p = 0.03) and muscle pain (17% (69/396) vs. 12% (39/317), OR 1.7 (95% CI 1.07-2.5), p = 0.03), but a lower frequency of gastrointestinal symptoms (7% (26/396) vs. 11% (35/317), OR 0.6 (95% CI 0.3-0.9), p = 0.04) compared with the 317 influenza testing-negative patients (S2 Table).

### Diagnosis concordance between Ag and AI testing

The concordance rate for diagnosing influenza between Ag and AI testing was evaluated. Among the 713 COVID-19 Ag-negative cases, AI testing showed an overall percent agreement of 62% (95% CI 58–66%) (442/713), a positive percent agreement of 47% (95% CI 40–53%) (125/269), and a negative percent agreement of 71% (95% CI 67–76%) (317/444) with Ag testing (S3 Table). The area under the receiver operating characteristic curve (AUROC) for AI testing, using Ag testing as the reference, was 0.59 (95% CI, 0.55–0.63), with a specificity of 0.71 and a sensitivity of 0.47 (S1 Fig). In analyses stratified by age and time from symptom onset, a higher frequency of disagreement between AI and Ag testing was observed among paediatric patients (<15 years) than among young and middle-aged adults (15–64 years): 43% (169/397) vs. 32% (91/284), OR 1.6 (95% CI 1.1-2.2), p = 0.006. Specifically, compared with adult patients, paediatric patients showed a higher frequency of negative disagreement (AI+ and Ag-) results relative to negative agreement (AI- and Ag-) results: 38% (98/256) vs. 17% (28/168), OR 3.1 (95% CI 1.9-5.0), p < 0.001. A similar trend was observed when comparing paediatric patients with elderly adults (≥65 years): 38% (98/256) vs. 0.5% (1/20), OR 12 (95% CI 1.6-89), p = 0.002. Among the 269 Ag-positive cases (although 270 were originally reported in Table 1, one patient with dual infection—COVID-19 Ag+ and influenza B Ag + —was excluded from the analyses; this patient is listed as COVID-19 Ag+ and Influenza Ag + , n = 1 in Fig 1), no significant difference in AI concordance was observed between strain A (49% (66/134)) and strain B (44% (59/135), OR 1.3 (95% CI 0.8-2.0), p = 0.4). These findings indicate that paediatric patients exhibited a higher frequency of discordant results between AI and Ag testing, characterized by a greater proportion of AI+ and Ag- results compared with adult patients.

### Influenza vaccination and testing positivity

We next analyzed whether influenza vaccination prior to testing affected differences in test positivity between AI and Ag testing. There was no significant difference in vaccination rates between AI+ and Ag+ cases: 21% (53/252) in AI+ patients vs. 23% (62/269) in Ag+ patients (OR 0.9 (95% CI 0.6-1.4), p = 0.6). Similarly, vaccination rates did not differ between AI+ and Ag- patients and AI- and Ag+ patients: 21% (27/127) vs. 25% (36/144), respectively (OR 0.8 (95% CI 0.5-1.4), p = 0.5) (S4 Table). These findings suggest that a history of influenza vaccination prior to testing did not influence differences in test positivity between the two methods.

### Impact of examination timing on influenza test positivity: Differences in time from symptom onset to testing among influenza testing-positive cases

**Fig 2:**
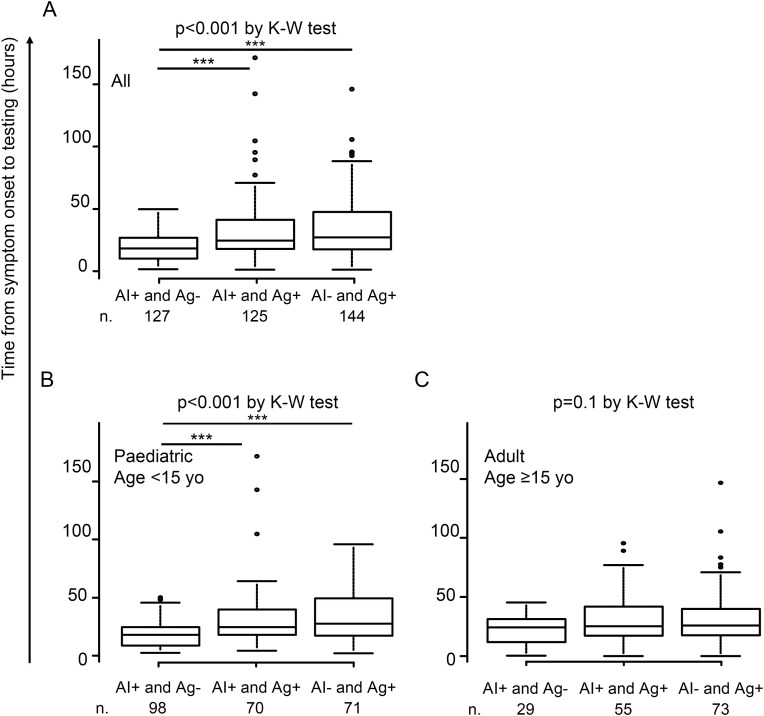
Difference in time from symptom onset to testing among influenza testing positives. Differences in time from symptom onset to testing among AI+ and Ag- patients, AI+ and Ag+ patients, and AI- and Ag+ patients, **(A)** in all, **(B)** paediatric, and **(C)** adult patients are shown. Kruskal-Wallis (K-W) test was conducted with post-hoc analysis using the Steel-Dwass test (***; p < 0.001).

**Fig 3:**
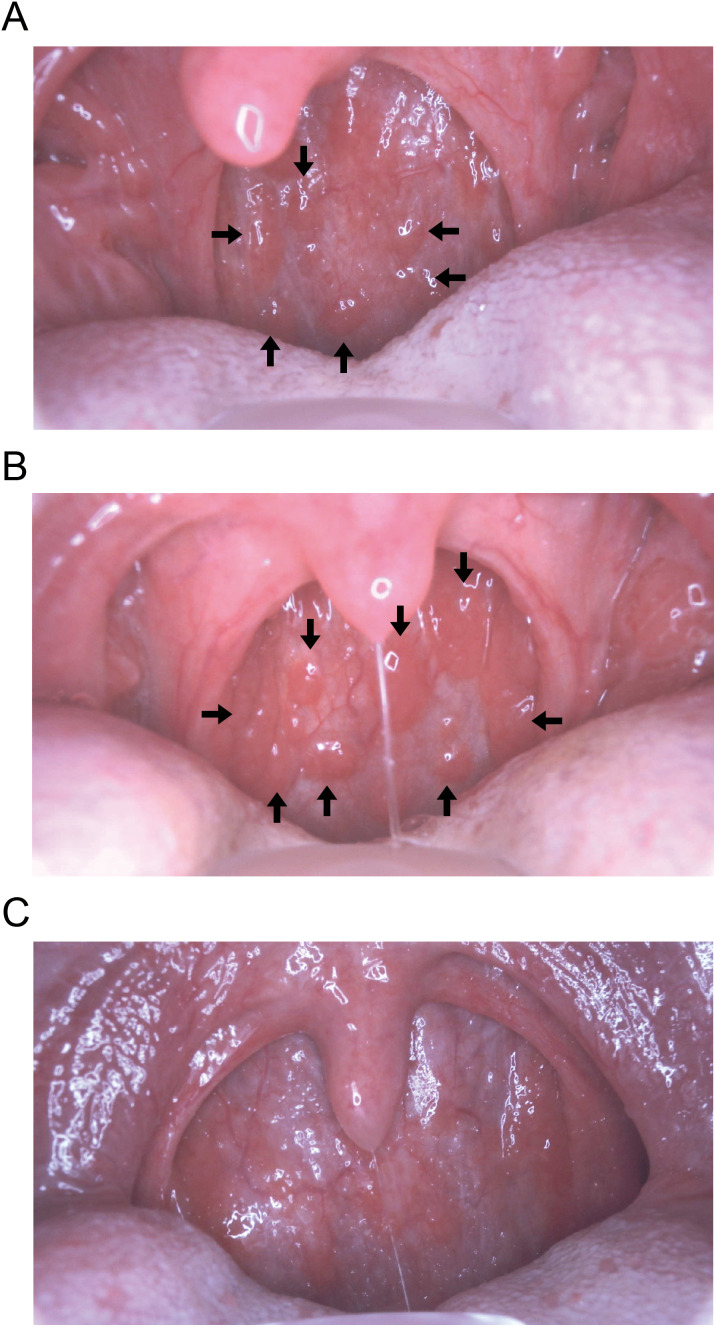
Posterior pharyngeal wall images in influenza infection. Representative pharyngeal images for each AI and Ag test result pair are shown. **(A)** The presence of Ikura (follicular) nodules (arrows) on the posterior pharyngeal wall is observed in AI+ and Ag- cases, **(B)** fusion of nodules (arrows) in AI+ and Ag+ cases, and **(C)** disappearance of nodules in AI- and Ag+ cases.

We analyzed differences in the time from symptom onset to testing among influenza-testing positive cases. Among the 396 COVID-19 Ag-negative and influenza-testing positive patients, significant differences in testing time were observed among AI+ and Ag- patients (n = 127, median 18 hours (IQR 10–27)), AI+ and Ag+ patients (n = 125, median 24 hours (IQR 18–41)), and AI- and Ag+ patients (n = 144, median 27 hours (IQR 17–47)) (p < 0.001 by Kruskal-Wallis test; p < 0.001 between AI+ and Ag- patients vs. AI+ and Ag+ patients, and between AI+ and Ag- patients vs. AI- and Ag+ patients by Steel-Dwass test) (Fig 2A). This difference remained significant among paediatric patients (<15 years old) (Fig 2B) but not among adults (age ≥ 15 years old) (Fig 2C). Representative pharyngeal images for each AI and Ag test result pair are shown in Fig 3: the presence of Ikura (follicular) nodules on the posterior pharyngeal wall in AI+ and Ag- cases (Fig 3A), fusion of nodules in AI+ and Ag+ cases (Fig 3B), and disappearance of nodules in AI- and Ag+ cases (Fig 3C). These findings suggest that AI-powered diagnostic systems may have an advantage in earlier detection after symptom onset compared with Ag testing, particularly among paediatric patients.

### Patient background and symptomatic differences between AI-positive and Ag-positive cases

We next analyzed differences in patient backgrounds and symptoms between those with AI+ and Ag- patients (n = 127) and those with AI- and Ag+ patients (n = 144). Compared with AI- and Ag+ patients, AI+ and Ag- patients were more frequently paediatric (OR 3.4 (95% CI 1.6-7.2), p = 0.001), tested earlier after symptom onset (<24 hours) (OR 2.6 (95% CI 1.3-4.9), p = 0.005), had a contact history with infected individuals (OR 4.6 (95% CI 2.2-9.3), p < 0.001), and more commonly presented with cough (OR 11 (95% CI 4.7-27), p < 0.001) and fever (≥38.0°C) (OR 5.6 (95% CI 2.8-11), p < 0.001). In contrast, they had a lower frequency of gastrointestinal symptoms (OR 0.2 (95% CI 0.05-0.9), p = 0.04) (Table 2). In subgroup analyses stratified by age (paediatric <15 years; adult ≥15 years) and testing time (early <24 hours; late ≥24 hours), the higher frequencies of paediatric age, early testing, contact history, and cough and fever among AI+ and Ag- patients remained significant in the paediatric and early-testing subgroups (S5 Table). However, in adults, the significance of early testing (OR 1.2 (95% CI 0.4–3.9), p = 0.8), contact history (OR 2.1 (95% CI 0.6-7.5), p = 0.3), and cough (OR 5.0 (95% CI 0.9-28), p = 0.07) was no longer observed. In the late-testing subgroup, the higher frequency of paediatric cases also became non-significant (OR 2.9 (95% CI 0.9–9.3), p = 0.07). Conversely, AI+ and Ag- patients exhibited lower frequencies of gastrointestinal symptoms in the paediatric subgroup (OR 0.03 (95% CI 0.001-0.5), p = 0.02) and lower frequencies of runny nose in the early-testing subgroup (OR 0.2 (95% CI 0.07-0.6), p = 0.006). These findings suggest that the AI system was more likely to yield positive influenza diagnoses in younger patients or those tested earlier after symptom onset—particularly among individuals with a contact history, cough, or fever—compared with Ag testing.

**Table 2:** Differences in patient background and symptom frequencies between influenza AI positives (AI+ and Ag-) and Ag positives (AI- and Ag+). Results from multivariable binary logistic regression analysis are shown.

n = 271 COVID-19 Ag-	Influenza	Yes	No	OR (95% CI)^a^	p
Sex (female)	AI+ and Ag-	67	60	1.5 (0.8–3.0)	0.2
	AI- and Ag+	65	79		
Age (<15 yo)	AI+ and Ag-	98	29	3.4 (1.6–7.2)	0.001
	AI- and Ag+	71	73		
Time from symptom onset to testing (<24 hrs)	AI+ and Ag-	84	43	2.6 (1.3–4.9)	0.005
	AI- and Ag+	63	81		
Contact with infected individuals	AI+ and Ag-	66	61	4.6 (2.2–9.3)	<0.001
	AI- and Ag+	49	95		
Use of antipyretics	AI+ and Ag-	47	80	0.8 (0.4–1.5)	0.5
	AI- and Ag+	73	71		
Influenza vaccination	AI+ and Ag-	27	100	0.7 (0.3–1.5)	0.4
	AI- and Ag+	36	108		
Symptom					
Cough	AI+ and Ag-	112	15	11 (4.7–27)	<0.001
	AI- and Ag+	91	53		
Headache	AI+ and Ag-	87	40	1.9 (0.9–3.7)	0.06
	AI- and Ag+	76	68		
Sore throat	AI+ and Ag-	86	41	1.6 (0.8–3.2)	0.2
	AI- and Ag+	70	74		
General fatigue	AI+ and Ag-	67	60	0.8 (0.4–1.5)	0.5
	AI- and Ag+	82	62		
Runny nose	AI+ and Ag-	73	54	0.6 (0.3–11)	0.09
	AI- and Ag+	91	53		
Fever (≥38.0°C)	AI+ and Ag-	96	31	5.6 (2.8–11)	<0.001
	AI- and Ag+	62	82		
Chill	AI+ and Ag-	50	77	1.7 (0.8–3.5)	0.2
	AI- and Ag+	44	100		
Appetite loss	AI+ and Ag-	30	97	0.8 (0.4–1.9)	0.7
	AI- and Ag+	43	101		
Joint pain	AI+ and Ag-	22	105	0.8 (0.3–2.1)	0.6
	AI- and Ag+	33	111		
Muscle pain	AI+ and Ag-	15	112	0.7 (0.3–1.9)	0.5
	AI- and Ag+	30	114		
Sweating	AI+ and Ag-	11	116	2.4 (0.7–8.5)	0.2
	AI- and Ag+	11	133		
Gastrointestinal symptom	AI+ and Ag-	4	123	0.2 (0.05–0.9)	0.04
	AI- and Ag+	12	132		

^a^ OR (95% CI); Odds ratio (95% confidence interval).

### Misdiagnosis by AI: False positive by AI for influenza in COVID-19 cases

We next investigated misdiagnosed cases by AI. Among the 100 COVID-19 Ag-positive cases (shown on the right side of the participant flow diagram in Fig 1), one case had dual infection (COVID-19 Ag+ and influenza Ag B+). Among the remaining 99 influenza Ag-negative cases, 77% (76/99) were negative by both influenza AI and Ag (AI- and Ag-), whereas 23% (23/99) were negative by Ag but positive by AI (AI+ and Ag-). PCR testing confirmed that all AI+ and Ag- cases were COVID-19 positive and influenza negative, indicating that these were false-positive diagnoses by the AI system. In comparisons between AI false-positive cases (n = 23) and AI-negative cases (n = 76), higher frequencies of cough (OR 28 (95% CI 2.5-327), p = 0.007) and fever (≥38.0°C) (OR 16 (95% CI 2.4-100), p = 0.004) were observed among the AI false positives (Table 3).

**Table 3:** Differences in patient background and symptom frequencies between influenza AI false positives and AI negatives. Results from multivariable binary logistic regression analysis are shown.

n = 99 COVID-19 Ag+	Influenza	Yes	No	OR (95% CI)^a^	p
Sex (Female)	AI false+	12	11	2.0 (0.4–9.6)	0.4
	AI-	42	34		
Age (<15 yo)	AI false+	3	20	2.1 (0.1–33)	0.6
	AI-	8	68		
Time from symptom onset to testing (<24hrs)	AI false+	11	12	1.1 (0.2–5.5)	0.9
	AI-	38	38		
Contact with infected individuals	AI false+	13	10	5.3 (0.9–30)	0.06
	AI-	35	41		
Use of antipyretics	AI false+	17	6	4.6 (0.7–29)	0.1
	AI-	34	42		
Influenza vaccination	AI false+	9	14	0.7 (0.2–2.8)	0.6
	AI-	30	46		
Symptom					
Cough	AI false+	21	2	28 (2.5–327)	0.007
	AI-	48	28		
Sore throat	AI false+	18	5	0.3 (0.04–2.1)	0.2
	AI-	59	17		
Headache	AI false+	15	8	3.7 (0.7–20)	0.1
	AI-	32	44		
General fatigue	AI false+	17	6	0.4 (0.05–3.9)	0.4
	AI-	47	29		
Runny nose	AI false+	11	12	0.3 (0.07–1.4)	0.1
	AI-	43	33		
Fever (≥38.0°C)	AI false+	10	13	16 (2.4–100)	0.004
	AI-	13	63		
Chill	AI false+	9	14	3.6 (0.7–17)	0.1
	AI-	17	59		
Appetite loss	AI false+	9	14	1.1 (0.2–6.7)	0.9
	AI-	21	55		
Joint pain	AI false+	9	14	0.7 (0.1–4.5)	0.7
	AI-	25	51		
Muscle pain	AI false+	5	18	2.7 (0.3–24)	0.4
	AI-	10	66		
Sweating	AI false+	10	13	5.7 (0.8–39)	0.07
	AI-	11	65		
Gastrointestinal symptom	AI false+	2	21	0.3 (0.02–5.3)	0.4
	AI-	6	70		

^a^ OR (95% CI); Odds ratio (95% confidence interval).

**Fig 4:**
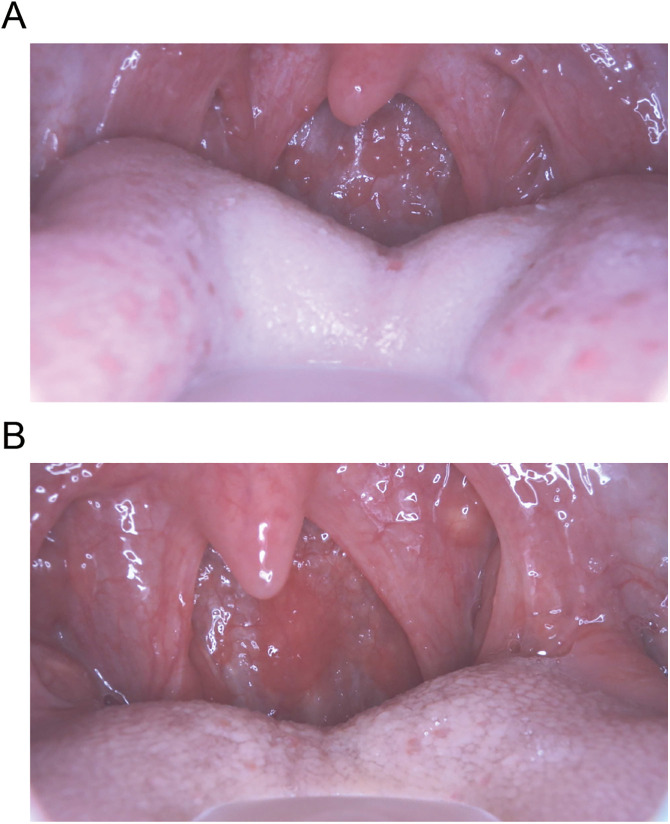
Misdiagnosis by AI. Although rapid antigen tests showed COVID-19 positive and influenza negative results, AI misdiagnosed the cases as influenza positive. **(A)** In Case 1, Ikura (follicular) nodules were observed on the posterior pharyngeal wall, while (B) in Case 2, round redness was noted.

Representative pharyngeal images of COVID-19-positive patients misdiagnosed as influenza-positive by AI are shown in Fig 4. Case 1: A 9-year-old male presented 21 hours after symptom onset with a body temperature of 36.9°C, headache, cough, sore throat, general fatigue, chill, and no known contact history with infected individuals. Multiple follicular nodules were observed on the posterior pharyngeal wall (Fig 4A).

Case 2: A 57-year-old male presented 17 hours after symptom onset with a body temperature of 38.8°C, headache, cough, sore throat, runny nose, general fatigue, sweating, and no known contact history with infected individuals. Round redness was observed on the posterior pharyngeal wall (Fig 4B).

These findings suggest that COVID-19 patients with prominent respiratory and systemic symptoms may exhibit pharyngeal findings similar to those of influenza virus infection, making it challenging for AI to accurately distinguish between the two viruses.

Finally, a summary of the differences in patient backgrounds and symptoms between AI-positive and Ag-positive cases is presented in Table 4. When compared with variables associated with crude influenza test positivity (AI+ or Ag+), summarized in S2 Table, younger age, cough, fever, and contact history were consistently associated with higher frequencies among AI-positive cases, as shown in Table 4. In contrast, runny nose was consistently associated with higher frequencies among Ag-positive cases. These findings highlight how patient background and symptomatic characteristics were strongly associated with test positivity for each diagnostic modality.

**Table 4:** Summary of differences in patient background and symptoms between AI-positive and Ag-positive cases.

	Higher frequencies in AI+	Higher frequencies in Ag+
Diagnosis	Host immune response-based	Pathogen viral load-based
Age	Paediatric (<15 yo)	Adult (≥15 yo)
Time	Early time (<24 hrs)	Late time (≥24 hrs)
Symptom	Cough	Gastrointestinal symptom
	Fever	Runny nose during early time
History	Contact history	
Risk	False negative in late time	False negative in early time
	False positive by other viral infection	

## Discussion

This cross-sectional study investigated the differences in patient backgrounds and symptoms between individuals who tested positive using a conventional rapid antigen (Ag) test and those who tested positive using a novel AI-powered pharyngeal endoscopy system. Compared with Ag-positive cases, AI-positive cases were more frequently paediatric patients, were tested earlier after symptom onset, were more likely to have had contact with infected individuals, and exhibited more pronounced symptoms. Additionally, this study identified several instances of AI misdiagnosis, particularly cases in which COVID-19–positive patients were incorrectly diagnosed by the AI system as having influenza virus infection.

In recent years, AI systems have been extensively developed and widely applied in the medical field, especially in image-based diagnostics. For example, AI has been incorporated into gastroendoscopy for cancer detection, assessment of invasion depth, and prediction of pathological diagnoses [7]. The novel AI-powered pharyngeal endoscopy system examined in this study diagnoses influenza virus infection by analyzing mucosal lesions—such as follicular nodules on the posterior pharyngeal wall and swelling of the lateral pharyngeal bands—in combination with clinical information [5]. Follicular nodules on the posterior pharyngeal wall, commonly referred to as “influenza follicles” or “Ikura follicles” (where ikura refers to fish caviar, derived from the Russian икра and Japanese ikura), typically appear in the early stage of infection as part of the host immune response to viral invasion, approximately eight hours after symptom onset [6]. In contrast, the sensitivity of rapid antigen testing increases over time, particularly after 24 hours from symptom onset [8,9], which is later than the appearance of follicular nodules. Consistent with this, in our study, the interval from symptom onset to testing was shorter among AI-diagnosed positive cases than among Ag-diagnosed positive cases. These findings underscore the importance of selecting diagnostic methods based on the time elapsed since symptom onset: AI testing may be more suitable for patients examined within 24 hours of symptom onset, whereas Ag testing may be more appropriate for those tested 24 hours or more after onset.

The higher frequency of paediatric patients among AI test positives compared with Ag test positives likely reflects differences in the underlying diagnostic mechanisms. Whereas Ag tests detect infection based on pathogen viral load, the AI system identifies infection through host immune responses. Age-related differences in immune function may therefore influence diagnostic outcomes. Thymic involution is a key age-related change, leading to reduced naïve T-cell production and a consequent decline in immune responsiveness to novel antigens [10,11]. This decline, known as *immunosenescence*, contributes to reduced vaccine efficacy and milder side effects in older adults compared with younger individuals [12–15]. The higher proportion of paediatric patients among AI test positives may thus reflect stronger immune responses to influenza virus infection in this group, resulting in more prominent mucosal lesions such as follicular nodules and more severe respiratory and systemic symptoms (e.g., cough and fever), thereby increasing the likelihood of a positive AI diagnosis. Previous studies identifying cough and fever as strong predictors of influenza infection support this interpretation [16,17]. In terms of immune responses and infection diagnosis, vaccination history was not identified as a significant factor associated with differences in test positivity between AI and Ag testing in this study. Humoral and cellular immunity acquired through vaccination may respond to viral infection at an early stage, thereby limiting both viral load and host immune responses, which could consequently reduce test positivity for both methods. Indeed, vaccination history was ranked as the least influential variable associated with test positivity in this AI model, contributing less than any respiratory or systemic symptom [5]. Furthermore, a recent study reported that high-dose influenza vaccination reduced disease severity compared with standard-dose vaccination [18]. Influenza infection following high-dose vaccination may therefore be associated with lower test positivity across both testing modalities.

Beyond age, timing of testing, and symptom profile, a history of contact with infected individuals was also more frequent among AI positives than Ag positives. This may be partly attributable to the AI model itself. In the original report on the development of this AI-powered endoscopy system, pharyngeal images were the most influential variable for diagnostic prediction, followed by body temperature, cough, and contact history [5]. Thus, the weighting of contact history as an important diagnostic feature may have contributed to its higher prevalence among AI positives in this study. Similarly, during the COVID-19 pandemic, contact history was reported as a significant predictor of test positivity in drive-through PCR testing [19]. These findings suggest that incorporating information on contact history is crucial for improving AI-based diagnostic accuracy through deep learning. In this study, the diagnostic performance of the AI model for Ag-positive cases showed an AUROC of 0.59, with a sensitivity of 47% and a specificity of 71%. These results indicate the presence of diagnostic discrepancies between the two tests. Implementing novel AI-based influenza testing that accounts for patient age, time from symptom onset, symptoms, and contact history may enhance point-of-care diagnostics in outpatient settings, particularly by complementing the moderate sensitivity (40%–80%) of conventional Ag testing [20].

Another notable finding was the occurrence of AI misdiagnoses. Pharyngitis can be caused by a range of pathogens, and the host immune response to these pathogens can result in similar follicular nodules on the posterior pharyngeal wall. Pathogens such as influenza virus, SARS-CoV-2, rhinovirus, enterovirus, and Mycoplasma pneumoniae have all been reported to cause such findings [21–24]. Moreover, COVID-19-infected patients have been observed to exhibit aggregated, millet-sized white spots surrounded by redness or ulcerative lesions on the posterior pharyngeal wall [25]. Among COVID-19 variants, the Omicron strain shows higher replication in the upper respiratory tract compared with earlier strains [26]. and has been associated with a higher frequency of sore throat than pre-Omicron variants [19,27]. During the study period, Omicron was the predominant strain; thus, the presence of follicular nodules and areas of redness in COVID-19 cases may have led to AI misdiagnosis as influenza virus infection. In the field of infectious diseases, AI has been extensively studied and applied to anti-infective drug discovery and antigen selection for vaccine development [28]. However, unlike drug discovery applications, the use of AI for infectious disease diagnostics remains challenging. Advancing AI-based diagnostic systems will require further collaboration between AI engineers and medical staff [29]. In this study, for instance, improving pathogen differentiation will likely require more advanced deep learning techniques that integrate imaging and clinical data from a variety of respiratory pathogens with similar symptoms, together with regional and seasonal epidemiological information.

Beyond diagnostic accuracy, the introduction of AI systems for point-of-care testing may help alleviate the burden associated with nasal swab collection required for Ag testing, thereby reducing both patient discomfort (e.g., nasal pain) and healthcare worker exposure risk [30]. Furthermore, diversifying point-of-care diagnostic tools beyond Ag kits would enhance preparedness for future pandemics by mitigating the risk of supply shortages of medical materials—such as Ag test kits—a challenge that was experienced globally during the COVID-19 pandemic [31].

This study has several limitations. First, influenza infection was not confirmed by PCR testing, introducing a potential risk of false-positive or false-negative results in both Ag and AI testing. Indeed, the reported diagnostic performance of the AI model for PCR-confirmed influenza infection includes an AUROC of 0.90, with sensitivity and specificity of 76% and 88% respectively [5]. Therefore, evaluation of AI and Ag test results in PCR-confirmed cases is warranted to more accurately assess diagnostic differences between the two tests. Second, immunological background information was not analyzed as a potential confounding factor. Given that the AI system operates on the basis of host immune responses, such background factors may influence AI test performance. For example, previous studies have reported stronger vaccine-related adverse reactions among individuals with autoimmune disorders or allergy histories following mRNA COVID-19 vaccination [13]. Incorporating immunological background information as an additional input may further refine and improve the diagnostic accuracy of AI systems. Third, the study population was predominantly paediatric (median 14 years, with relatively few elderly participants (adult median age 36.5 years). Further studies including a larger number of elderly patients are warranted to better assess the impact of age and to confirm the potential advantages of AI-based diagnosis leveraging host immune responses in paediatric populations. Finally, because of the limited sample size for analyses, several variables showed statistically significant associations accompanied by wide 95% confidence intervals—such as cough and fever in Table 3—indicating the need for cautious interpretation.

In conclusion, this study highlights the differences in patient backgrounds and symptoms between cases testing positive by conventional rapid Ag assays and those testing positive by a novel AI-powered pharyngeal endoscopy system. The introduction of this AI-based system into point-of-care influenza diagnostics—particularly for patients tested shortly after symptom onset, paediatric patients, symptomatic individuals with fever or cough, and those with a history of contact with infected persons—may enhance diagnostic performance and help compensate for the limited sensitivity of conventional rapid Ag testing. Understanding the diagnostic mechanisms underlying each test will facilitate appropriate selection between Ag and AI testing, thereby supporting more accurate point-of-care diagnostics. Furthermore, this study underscores the potential of AI-powered medical diagnostic systems to advance pathogen differentiation in pharyngitis, including influenza and COVID-19.

## Materials and methods

### Ethics statement

This study was approved by the ethical review boards of Sasebo Memorial Hospital and Moriyama Clinic Ethics Committee, Japan (approval number 2023-09). The research involving human data use complied with all relevant national regulations and institutional policies and was conducted in accordance with the principles outlined in the Declaration of Helsinki. Written informed consent was obtained from all participants; for participants aged <18 years, consent was obtained from a parent or legal guardian.

### Subjects and data collection

Patients aged five years or older who underwent nasopharyngeal swab-based rapid antigen testing for influenza virus and COVID-19 were recruited from Sasebo Memorial Hospital and Moriyama Clinic between February 2024 and March 2025. Rapid antigen tests were performed using kits from multiple manufacturers that are approved for clinical diagnosis and routinely used as point-of-care tests in outpatient settings. Patients younger than five years of age or those unable to comply with instructions for pharyngeal image acquisition required for AI testing were excluded from the study. A total of 864 patients were registered in the study. Among them, 51 patients (5.9%) failed to complete the AI-based influenza test because the AI system was unable to recognize the pharyngeal images. Thus, 813 patients had valid results for both Ag and AI testing. Because patient background and symptom information were collected as mandatory inputs in the AI testing system prior to image acquisition, no data were missing for these variables, and all were used in the analyses for this study. In cases where the AI system diagnosed influenza infection in patients who were positive for COVID-19 antigen and negative for influenza antigen (COVID-19 Ag + , influenza Ag-, and influenza AI+), confirmatory PCR testing for COVID-19 and influenza viruses was conducted using swab specimens at a commercial laboratory (Genesis Healthcare, Tokyo, Japan).

### AI system

The AI-powered pharyngeal endoscopy system for influenza diagnosis was originally developed and reported by Okiyama et al. [5]. Briefly, the system operates as follows: (1) Input of background and symptom information: Patient background and symptom data—including sex, age, time of first symptom onset, history of contact with infected individuals, use of antipyretics, and influenza vaccination status—are collected at the outpatient visit and entered into the AI system as mandatory fields prior to testing. (2) Pharyngeal image acquisition: To ensure image quality, rapid continuous shooting (18 images over 3.0 seconds) is performed to minimize motion blur. A light-emitting diode light source and a disposable clear camera cover are used to depress the tongue and prevent fogging from exhalation. The viewing angle is appropriately restricted to reduce image distortion. The system automatically recognizes the posterior pharyngeal wall; if recognition fails, image acquisition is repeated. (3) AI-based diagnosis: The diagnostic AI model was developed using pharyngeal images and background/clinical information from PCR-confirmed influenza cases. The model is an ensemble comprising a multimodal convolutional neural network (MM-CNN), a multi-view convolutional neural network (MV-CNN), and boosting models. Data from 7,831 participants in the training stage and 659 participants in the validation stage, collected from hospitals in Japan, were used for model development. Pharyngeal images were ranked as the most influential predictors of influenza diagnosis, followed by body temperature and cough. The reported diagnostic performance of the AI model for PCR-confirmed influenza infection includes an AUROC of 0.90, with sensitivity and specificity of 76% and 88%, respectively.

### Statistical analysis

Statistical analyses were performed using EZR (developed by Jichi Medical University, Saitama, Japan) [32]. To minimize bias in symptom assessment attributable to COVID-19 infection, analyses of influenza test positivity were restricted to COVID-19 Ag- cases. Differences in symptom frequencies between influenza testing-positive and influenza testing-negative cases were assessed using Fisher’s exact test. The sensitivity and specificity of the AI system relative to Ag test results were evaluated using the AUROC. The time from symptom onset to testing among the AI+ and Ag-, AI+ and Ag + , and AI- and Ag+ groups was analyzed using the Kruskal-Wallis test, followed by the Steel-Dwass test for post-hoc comparisons. Multivariate binary logistic regression was conducted to identify patient background factors and symptoms associated with (a) AI positives (AI+ and Ag-) versus Ag positives (AI- and Ag+), and (b) false-positive AI results (AI false+) versus AI negatives (AI-) among COVID-19–infected patients. Patient background and symptom variables included in the multivariate models were those collected as mandatory input information for AI testing. To assess multicollinearity among the independent variables, variance inflation factors (VIFs) were calculated, and no variable exceeded a VIF of 5 (S6 Table). Calibration was evaluated using the Hosmer-Lemeshow goodness-of-fit test, which showed no significant lack of fit (p ≥ 0.05) in each test (S6 Table).

## Supporting information

S1 Table Study dataset. (XLSX)

S2 Table Differences in background and symptom frequencies between influenza testing positives (AI+ or Ag+) and negatives (AI- and Ag-). (XLSX)

S3 Table Concordance between antigen (Ag) and artificial intelligence (AI) testing for influenza. P values by Fisher’s exact tests are shown.
(XLSX)

S4 Table Difference in influenza vaccination frequency between AI positives and Ag positives. P values by Fisher’s exact tests are shown.
(XLSX)

S5 Table Subgroup analyses of differences in background and symptom frequencies between influenza AI positives (AI+ and Ag-) and Ag positives (AI- and Ag+). Results from multivariable binary logistic regression analysis are shown.
(XLSX)

S6 Table Variance inflation factor (VIF) scores and Hosmer–Lemeshow goodness-of-fit test results for each binary logistic regression analysis. (XLSX)

S1 Fig Sensitivity and specificity of AI testing relative to Ag testing. Receiver operating characteristic (ROC) curve and the area under the curve (AUC) with 95% confidence intervals are shown.
(TIF)

## References

[1] Takahashi H, Nagamatsu H, Yamada Y, Toba N, Toyama-Kousaka M, Ota S, et al. Surveillance of seasonal influenza viruses during the COVID-19 pandemic in Tokyo, Japan, 2018-2023, a single-center study. Influenza Other Respir Viruses. 2024;18(1):e13248. doi: 10.1111/irv.13248 38188373 PMC10767599

[2] Bajema KL, Bui DP, Yan L, Li Y, Rajeevan N, Vergun R, et al. Severity and long-term mortality of COVID-19, influenza, and respiratory syncytial virus. JAMA Intern Med. 2025;185(3):324–34. doi: 10.1001/jamainternmed.2024.7452 39869355 PMC11773409

[3] Shirley JD, Bennett SA, Binnicker MJ. Current regulatory landscape for viral point-of-care testing in the United States. J Clin Virol. 2023;164:105492. doi: 10.1016/j.jcv.2023.105492 37210882

[4] Pondaven-Letourmy S, Alvin F, Boumghit Y, Simon F. How to perform a nasopharyngeal swab in adults and children in the COVID-19 era. Eur Ann Otorhinolaryngol Head Neck Dis. 2020;137(4):325–7. doi: 10.1016/j.anorl.2020.06.001 32646750 PMC7274641

[5] Okiyama S, Fukuda M, Sode M, Takahashi W, Ikeda M, Kato H, et al. Examining the use of an artificial intelligence model to diagnose influenza: development and validation study. J Med Internet Res. 2022;24(12):e38751. 10.2196/38751 PMC9823578 36374004

[6] Miyamoto A, Watanabe S. Influenza follicles and their buds as early diagnostic markers of influenza: typical images. Postgrad Med J. 2016;92(1091):560–1. doi: 10.1136/postgradmedj-2016-134271 27466411 PMC5013091

[7] Okagawa Y, Abe S, Yamada M, Oda I, Saito Y. Artificial intelligence in endoscopy. Dig Dis Sci. 2022;67(5):1553–72. 34155567 10.1007/s10620-021-07086-z

[8] Chartrand C, Leeflang MMG, Minion J, Brewer T, Pai M. Accuracy of rapid influenza diagnostic tests: a meta-analysis. Ann Intern Med. 2012;156(7):500–11. doi: 10.7326/0003-4819-156-7-201204030-00403 22371850

[9] Watanabe M, Nakagawa N, Ito M, Ihara T. Sensitivity of rapid immunoassay for influenza A and B in the early phase of the disease. Pediatr Int. 2009;51(2):211–5. doi: 10.1111/j.1442-200X.2008.02696.x 19405918

[10] Gruver AL, Hudson LL, Sempowski GD. Immunosenescence of ageing. J Pathol. 2007;211(2):144–56. doi: 10.1002/path.2104 17200946 PMC1931833

[11] Goronzy JJ, Weyand CM. T cell development and receptor diversity during aging. Curr Opin Immunol. 2005;17(5):468–75. doi: 10.1016/j.coi.2005.07.020 16098723

[12] Mori M, Yokoyama A, Shichida A, Sasuga K, Maekawa T, Moriyama T. Impact of sex and age on vaccine-related side effects and their progression after booster mRNA COVID-19 vaccine. Sci Rep. 2023;13(1):19328. doi: 10.1038/s41598-023-46823-4 37935801 PMC10630308

[13] Mori M, Yokoyama A, Shichida A, Sasuga K, Maekawa T, Moriyama T. Impact of sex and age on mRNA COVID-19 vaccine-related side effects in Japan. Microbiol Spectr. 2022. doi: 10.1128/spectrum.01309-22 PMC9769945 36314943

[14] Mori M, Doi T, Murata M, Moriyama Y, Akino K, Moriyama T, et al. Impact of nutritional status on antibody titer after booster mRNA COVID-19 vaccine among elderly adults in Japan. J Infect Dis. 2024;229(4):1035–40. 37962870 10.1093/infdis/jiad495

[15] Goronzy JJ, Weyand CM. Understanding immunosenescence to improve responses to vaccines. Nat Immunol. 2013;14(5):428–36. doi: 10.1038/ni.2588 23598398 PMC4183346

[16] Monto AS, Gravenstein S, Elliott M, Colopy M, Schweinle J. Clinical signs and symptoms predicting influenza infection. Arch Intern Med. 2000;160(21):3243–7. doi: 10.1001/archinte.160.21.3243 11088084

[17] Ohmit SE, Monto AS. Symptomatic predictors of influenza virus positivity in children during the influenza season. Clin Infect Dis. 2006;43(5):564–8. doi: 10.1086/506352 16886147

[18] Pardo-Seco J, Rodríguez-Tenreiro-Sánchez C, Giné-Vázquez I, Mallah N, Mirás-Carballal S, Piñeiro-Sotelo M, et al. High-dose influenza vaccine to reduce hospitalizations. N Engl J Med. 2025;393(23):2303–12. doi: 10.1056/NEJMoa2509834 40888694

[19] Mori M, Yokoyama K, Sanuki R, Inoue F, Maekawa T, Moriyama T. Analyzing factors affecting positivity in drive-through COVID-19 testing: a cross-sectional study. Virol J. 2024;21(1):111. doi: 10.1186/s12985-024-02388-w 38745200 PMC11094999

[20] Uyeki TM, Hui DS, Zambon M, Wentworth DE, Monto AS. Influenza. Lancet. 2022;400(10353):693–706. 36030813 10.1016/S0140-6736(22)00982-5 PMC9411419

[21] Orzell S, Suryadevara A. Pharyngitis and pharyngeal space infections. In: Domachowske J, editor. Introduction to clinical infectious diseases: A problem-based approach. Cham: Springer International Publishing; 2019. pp. 53–66.

[22] Inui G, Tomita K, Yamasaki A. Cobblestone throat in a younger patient infected with the omicron variant of the SARS-CoV-2 virus. Am J Trop Med Hyg. 2023;109(2):221–2. doi: 10.4269/ajtmh.22-0747 37364864 PMC10397436

[23] Takahashi H. Variations in the appearance of posterior pharyngeal wall follicles in individuals with viral upper respiratory infections according to the virus and the stage of infection: A case series. Int J Infect Dis. 2022;119:140–1. doi: 10.1016/j.ijid.2022.03.056 35378263 PMC8975593

[24] Kenzaka T, Kyotani M, Goda K, Akita H. Reply to “Influenza follicles and their buds as early diagnostic markers of influenza: typical images” and demonstration of lymphoid follicles in the posterior pharyngeal walls of patients with mycoplasmal pneumonia. Postgrad Med J. 2018;94(1111):311–2. doi: 10.1136/postgradmedj-2017-135540 29440479 PMC5931247

[25] Yamashita T, Fukuchi T, Sugawara H. Appearance of a sore throat caused by the SARS-CoV-2 Omicron variant. J Gen Fam Med. 2022;24(2):129–30. 36718285 10.1002/jgf2.589 PMC9877946

[26] Suzuki R, Yamasoba D, Kimura I, Wang L, Kishimoto M, Ito J, et al. Attenuated fusogenicity and pathogenicity of SARS-CoV-2 Omicron variant. Nature. 2022;603(7902):700–5. doi: 10.1038/s41586-022-04462-1 35104835 PMC8942852

[27] Menni C, Valdes AM, Polidori L, Antonelli M, Penamakuri S, Nogal A, et al. Symptom prevalence, duration, and risk of hospital admission in individuals infected with SARS-CoV-2 during periods of omicron and delta variant dominance: a prospective observational study from the ZOE COVID Study. Lancet. 2022;399(10335):1618–24. doi: 10.1016/S0140-6736(22)00327-0 35397851 PMC8989396

[28] Wong F, de la Fuente-Nunez C, Collins JJ. Leveraging artificial intelligence in the fight against infectious diseases. Science. 2023;381(6654):164–70. doi: 10.1126/science.adh1114 37440620 PMC10663167

[29] Miglietta L, Rawson TM, Galiwango R, Tasker A, Ming DK, Akogo D. Artificial intelligence and infectious disease diagnostics: state of the art and future perspectives. Lancet Infect Dis. 2025. 10.1016/S1473-3099(25)00354-8 40972627

[30] Kaufman AC, Brewster R, Rajasekaran K. How to perform a nasopharyngeal swab - an otolaryngology perspective. Am J Med. 2020;133(11):1280–2. doi: 10.1016/j.amjmed.2020.05.004 32492374 PMC7261357

[31] Cornish NE, Bachmann LH, Diekema DJ, McDonald LC, McNult P, Stevens-Garcia J, et al. Pandemic demand for SARS-CoV-2 testing led to critical supply and workforce shortages in U.S. clinical and public health laboratories. J Clin Microbiol. 2023;61(7):e0318920. doi: 10.1128/jcm.03189-20 37070976 PMC10358151

[32] Kanda Y. Investigation of the freely available easy-to-use software “EZR” for medical statistics. Bone Marrow Transplant. 2013;48(3):452–8. doi: 10.1038/bmt.2012.244 23208313 PMC3590441

