# Supplementary figures and images for: Infectious disease diagnosis by artificial intelligence (AI): Differences in patient backgrounds and symptoms between antigen test positives and novel AI-powered pharyngeal endoscopy test positives
Source: PLOS Digit Health. 2026 Feb 11;5(2):e0001233. doi: 10.1371/journal.pdig.0001233 (PMC12893528; doi:10.1371/journal.pdig.0001233)

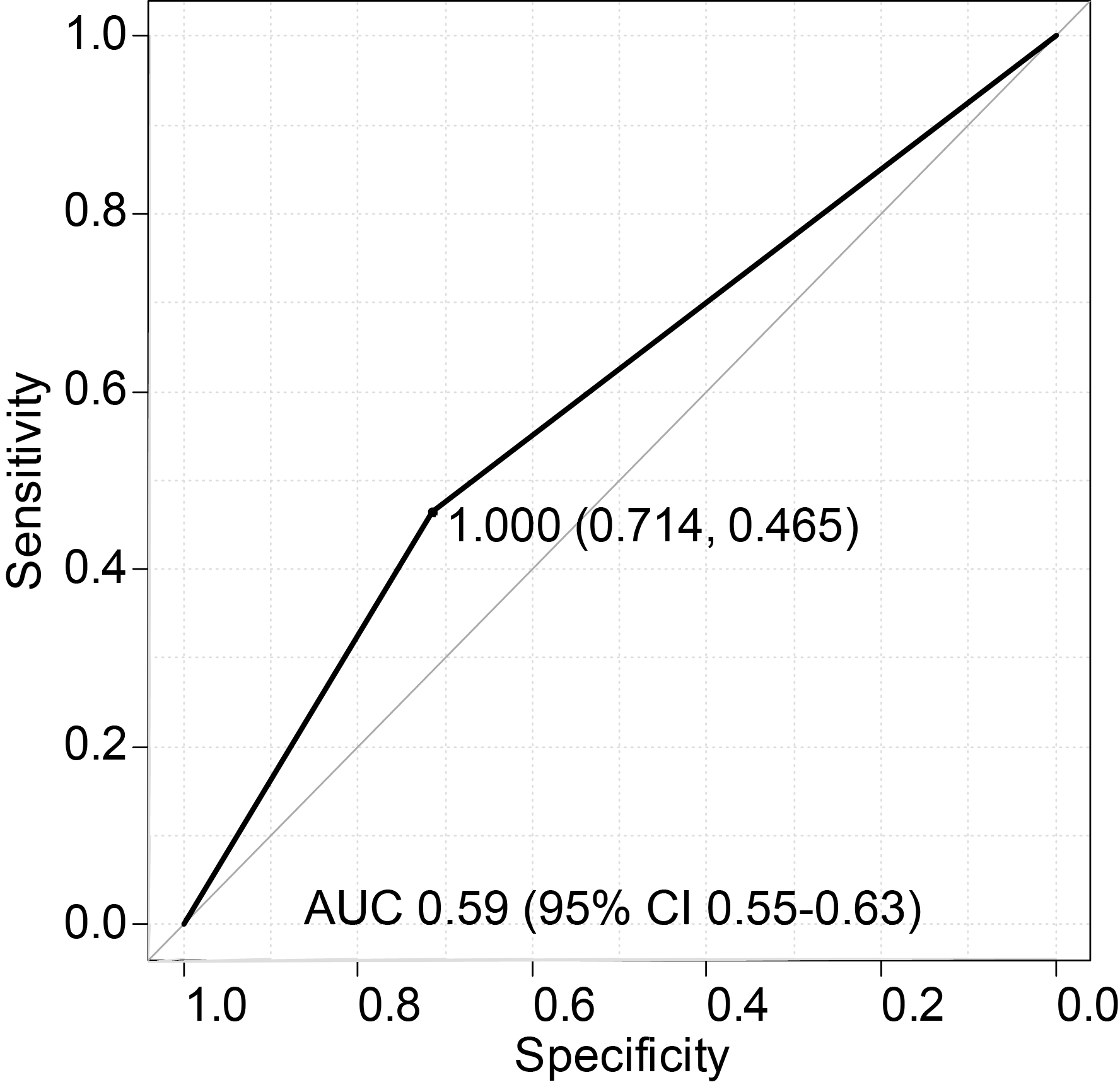

Supplement: S1 Fig — Receiver operating characteristic (ROC) curve and the area under the curve (AUC) with 95% confidence intervals are shown. (TIF) [file pdig.0001233.s007.tif]
